# Effects of Short-Term Depuration on Muscle Nutritional Quality and Flavor Composition in *Carassius auratus gibelio*

**DOI:** 10.3390/foods14234155

**Published:** 2025-12-03

**Authors:** Chuntao Xue, Duhuang Chen, Wenqiang Jiang, Yan Lin, Yanshun Xu, Lingzhan Xue, Siyue Lu, Haiping Fan, Xuegui Li, Linghong Miao

**Affiliations:** 1 Wuxi Fisheries College, Nanjing Agricultural University, No. 9 Shanshui East Road, Wuxi 214081, China; 2 Key Laboratory of Freshwater Fisheries and Germplasm Resources Utilization, Ministry of Agriculture and Rural Affairs, No. 9 Shanshui East Road, Wuxi 214081, China; 3 Freshwater Fisheries Research Institute of Fujian, Fuzhou 350002, China; 4 School of Food Science and Technology, Jiangnan University, Lihu 1800, Wuxi 214122, China

**Keywords:** *Carassius auratus gibelio*, short-term culture, salinity, muscle nutrition, quality

## Abstract

This study evaluated the effects of short-term depuration under different salinity levels on the muscle nutritional composition and quality of *Carassius auratus gibelio*, aiming to provide guidance for enhancing the value of farmed crucian carp. A total of 240 fish (450 ± 50 g) were reared in recirculating aquaculture systems at salinities of 0‰ (S0), 3‰ (S3), 6‰ (S6), and 9‰ (S9) for 0 (D0), 5 (D5), and 10 days (D10). Dorsal muscle samples were analyzed for proximate composition, amino acids, fatty acids, flavor-related nucleotides, geosmin, and volatile compounds. Short-term depuration significantly improved muscle protein content, total amino acids, and umami amino acids. Culturing at 6‰ salinity for 10 days enhanced crude protein, total amino acids, umami amino acids, and lipid indices associated with cardiovascular benefits, while 9‰ salinity for 5 days increased crude lipid, total umami amino acids content, and the essential amino acid index (EAAI). Geosmin content decreased under moderate salinity but tended to accumulate at higher salinities. Amino acid scoring identified lysine, methionine, and cysteine as limiting under certain conditions, indicating a need for supplementation. Overall, short-term salinity depuration effectively improves muscle nutritional composition, fatty acid profiles, and flavor attributes, offering a practical approach to producing higher-value *C. auratus gibelio* with enhanced health benefits and consumer appeal.

## 1. Introduction

Crucian carp (*Carassius auratus*) is one of the most important freshwater aquaculture species in China due to its broad diet, high production yield, strong environmental adaptability, and rich nutritional value. In 2023, the total production of crucian carp in China reached 2.84 million tons, serving as aquatic food [1]. Currently, crucian carp is primarily cultured in traditional ponds. However, issues such as high stocking densities, poor water exchange, and unbalanced feed formulations often result in reduced muscle quality and the accumulation of undesirable off-flavors [2,3]. Moreover, due to its benthic feeding behavior, crucian carp is especially prone to the muscle accumulation of secondary metabolites such as geosmin and 2-methylisoborneol, which produce earthy–musty off-flavors that substantially reduce the consumer acceptance of fish [4]. Therefore, developing effective short-term strategies to improve muscle quality and reduce off-flavor compounds in market-size crucian carp is of considerable practical importance for enhancing product quality and commercial value.

Recent studies have demonstrated that transferring market-size crucian carp into a controlled recirculating aquaculture system (RAS) subjected to precise water quality management, combined with intermittent fasting, can effectively improve muscle quality while reducing the accumulation of off-flavor compounds [5]. This approach, called depuration, does not adversely affect fish survival or health and has been reported to promote the excretion of metabolic waste, reduce fat deposition, increase muscle fiber density, and eliminate odor precursors [6,7]. Currently, two main strategies are employed to improve fish quality: depuration in freshwater and short-term depuration under saline conditions. Freshwater depuration primarily stimulates metabolic activity through continuous water flow, whereas depuration under saline conditions improves fish quality by inducing mild osmotic stress [8]. Compared to depuration in freshwater, depuration under saline conditions offers several advantages, including shorter treatment durations and reduced water consumption [9]. Short-term depuration under saline conditions has been shown to effectively eliminate surface pathogens, reduce earthy–musty odors, and improve muscle elasticity, thereby enhancing the overall sensory and nutritional quality of freshwater fish [10,11]. For example, grass carp subjected to 48 h of depuration at 3‰ salinity exhibited a significant increase in umami amino acid content in muscle [11]. Similarly, crucian carp exposed to low-salinity seawater (6‰) for 15 days showed a marked reduction in geosmin and 2-methylisoborneol concentrations [12]. In another study, largemouth bass cultured for 10 weeks at salinities of 3‰ and 6‰ exhibited a significant increase in muscle PUFA content [13].

This study was designed to systematically evaluate the effects of different salinity levels (0‰, 3‰, 6‰, and 9‰) and depuration durations (0, 5, and 10 days) on the muscle nutritional composition and quality of *Carassius auratus gibelio*. Particular attention was given to analyzing changes in the proximate composition, volatile flavor compounds, geosmin concentration, flavor-related nucleotides, free fatty acid profiles, and hydrolyzed amino acids. The objective was to provide theoretical support for the establishment of salinity-based depuration protocols aimed at improving the quality of crucian carp flesh and to support the standardization of best practices in commercial aquaculture.

## 2. Materials and Methods

### 2.1. Ethics Approval and Consent to Participate

All experimental protocols, methods, and feeding procedures were approved by the Institutional Animal Care and Use Committee of the Freshwater Fisheries Research Center, Chinese Academy of Fishery Sciences. All animal handling and experimental procedures were conducted in accordance with the approved ethical guidelines.

### 2.2. Short-Term Depuration Under Saline Conditions

Experimental *Carassius auratus gibelio* were obtained from Zhaoxing Fish Breeding Co., Ltd. (Shunchang County, Nanping, China). A total of 240 healthy adult fish (450 ± 50 g) of uniform size were sourced from the same culture pond. The fish were acclimated in concrete tanks and fed a commercial diet (Tongwei Co., Ltd., Wuxi, China) at 2% body weight per day for 7 days. Following acclimation, the fish were randomly assigned to 12 temperature-controlled circular tanks (3.0 m diameter, 1.2 m depth, 20 fish per tank). Four salinity levels were established using artificial sea salts: 0‰ (freshwater control, S0), 3‰ (S3), 6‰ (S6), and 9‰ (S9), with three replicate tanks per condition. No feed was provided during the experimental period. Each system was equipped with independent inflow and drainage, and continuous aeration was provided via microporous diffusers. The environmental parameters were maintained as follows: water temperature 25 ± 1 °C, dissolved oxygen ≥ 6 mg/L, and pH 7.0 ± 0.2.

### 2.3. Sample Collection and Preparation

Fish samples were collected from each treatment tank (S0, S3, S6, and S9) at 0 days (before depuration, D0), 5 days (D5), and 10 days (D10). At each sampling point, three fish were randomly selected from each tank. After rapid tail ablation and exsanguination, ~30 g of dorsal muscle (scaled and skinned) was excised from both sides of each fish. These muscle samples were homogenized and divided into two portions, one of which was stored at –20 °C for proximate composition analysis (moisture, crude protein, crude lipid, and ash). Meanwhile, the other portion was flash-frozen in liquid nitrogen and stored at –80 °C for analyses of hydrolyzed amino acids, free fatty acids, flavor-related nucleotides, volatile flavor compounds, and geosmin.

### 2.4. Determination of Proximate Composition

The moisture, crude protein, crude lipid and ash contents of the muscle were determined by the standard method of the Association of Official Analytical Chemists [14]. Moisture content was determined by drying muscle samples at 105 °C until constant weight. The ash content was determined via incineration in a muffle furnace at 550 °C until constant weight was achieved. Finally, the crude protein content was measured with the Kjeldahl method using an automatic Kjeldahl analyzer, while the crude lipid content was analyzed through Soxhlet extraction using anhydrous diethyl ether as the solvent.

### 2.5. Analysis of Amino Acid Composition and Nutritional Value

The hydrolyzed amino acid composition and content in muscle tissue were quantified following the standardized acid hydrolysis protocol (GB/T 18246) [15]. Specifically, 0.1 g of muscle sample was subjected to hydrolysis with 8 mL of 6 M sulfuric acid at 120 °C for 22–24 h under anoxic conditions maintained by a nitrogen environment. Post-hydrolysis, the mixture was neutralized using 4.8 mL of 10 M sodium hydroxide, adjusted to a final volume of 25 mL with ultrapure water, and filtered through dual-layer filter membranes. Subsequent quantification of individual amino acids was performed employing an automated amino acid analyzer (Agilent 1100, Santa Clara, CA, USA) [16]. Meanwhile, the nutritional value of amino acids was evaluated based on FAO/WHO amino acid scoring patterns and the egg protein model proposed by the Chinese Center for Disease Control and Prevention. The indices were calculated as follows:

$$\begin{array}{l} \mathrm{Amino}\;\mathrm{acid}\;\mathrm{score}\;(\mathrm{AAS})=\mathrm{AA}/\mathrm{FAO}\text{-}\;\mathrm{and}\;\mathrm{WHO}\text{-}\mathrm{recommended}\;\mathrm{content}\;\mathrm{of}\;\mathrm{the}\;\mathrm{EAA} \end{array}$$

Chemistry score (CS) = AA/Content of the EAA in egg protein

$$\mathrm{Essential}\;\mathrm{amino}\;\mathrm{acid}\;\mathrm{Index}\;(\mathrm{EAAI})=\sqrt[{n}]{\frac{100t1}{S1}\times \frac{100t2}{S2}\times \cdots \frac{100\mathrm{tn}}{\mathrm{Sn}}},$$

where AA is the essential amino acid content in the sample protein (mg/g×N):

$$\begin{array}{l} \mathrm{AA}=(\mathrm{EAA}\;\mathrm{content}\;\mathrm{in}\;\mathrm{fresh}\;\mathrm{sample}/\mathrm{Crude}\;\mathrm{protein}\;\mathrm{content}\;\mathrm{in}\;\mathrm{fresh}\;\mathrm{sample})\times 6.25\times 1000. \end{array}$$

$$\begin{array}{l} N=\mathrm{number}\;\mathrm{of}\;\mathrm{amino}\;\mathrm{acids};\;t1,\;t2\;\ldots \mathrm{tn}\;=\;\mathrm{ratios}\;\mathrm{of}\;\mathrm{EAAs}\;\mathrm{in}\;\mathrm{the}\;\mathrm{sample}\;\mathrm{protein}\\ (\mathrm{mg}/g\times N);\;\mathrm{and}\;S1,\;S2\;\ldots \mathrm{Sn}\;=\;\mathrm{ratios}\;\mathrm{of}\;\mathrm{EAAs}\;\mathrm{in}\;\mathrm{egg}\;\mathrm{protein}\;(\mathrm{mg}/g\times N). \end{array}$$

### 2.6. Analysis of Free Fatty Acids and Their Nutritional Quality

The composition and content of free fatty acids were analyzed by gas chromatography following lipid extraction and derivatization. Briefly, total lipids were extracted from muscle samples using a chloroform–methanol mixture (2:1, *v*/*v*). Fatty acid methyl esters (FAMEs) were prepared by transesterification with 14% boron trifluoride in methanol at 70 °C for 1 h. Separation was performed on an HP-88 capillary column (100 m × 0.25 mm × 0.20 μm) using helium as the carrier gas, with a temperature program starting at 140 °C (held for 5 min), ramping at 4 °C/min to 240 °C, and held for 15 min. Fatty acids were identified by comparing retention times with those of authentic standards, and quantification was achieved using internal standard (C19:0) calibration curves [17]. Nutritional assessments were conducted based on the peroxidizability index (PI), atherogenic index (AI), and thrombogenic index (TI). These parameters were calculated as follows:

PI = (C20:5n-3 + C22:6n-3)/C16:0

AI = (C12:0 + 4 × C14:0 + C16:0)/(∑PUFA n-6 + ∑PUFA n-3 + ∑MUFA)

$$\begin{array}{l} \mathrm{TI}=(C14:0\;+\;C16:0\;+\;C18:0)/[0.5\;\times \;\sum \mathrm{MUFA}\;+\;0.5\;\times \;\sum \mathrm{PUFA}\;n\text{-}6\;+\\ 3\;\times \;\sum \mathrm{PUFA}\;n\text{-}3\;+\;(\sum \mathrm{PUFA}\;n\text{-}3/\sum \mathrm{PUFA}\;n\text{-}6)] \end{array}$$

Note: C20:5n-3, EPA content; C22:6n-3, DHA content; C16:0, palmitic acid content; C12:0, lauric acid content; C14:0, myristic acid content; C18:0, stearic acid content; PUFA n-6, total content of n-6 series PUFAs; PUFA n-3, total content of n-3 series PUFAs; MUFA, total content of monounsaturated fatty acids.

### 2.7. Analysis of Flavor-Related Nucleotides

The analysis of flavor-related nucleotides was performed according to the following procedure. Muscle samples were thawed at 4 °C and precisely weighed (5.00 g) into a centrifuge tube. Then, 25 mL of a 5% perchloric acid solution was added, and the mixture was homogenized twice (20 s each) at 8000 r/min using a high-speed disperser in an ice bath. The homogenizer blade was rinsed twice with 10 mL of the same 5% perchloric acid solution, and the rinsates were combined into the centrifuge tube. The mixture was centrifuged at 4 °C and 1000 r/min for 10 min. The supernatant was collected, and the pellet was repeatedly washed three times with 10 mL of 5% perchloric acid. The combined supernatants were neutralized to pH 6.5 using NaOH, diluted to a final volume of 100 mL with high-purity water, and filtered through a 0.22 μm membrane. A 1 mL aliquot of the filtrate was then injected into an ultra-high-performance liquid chromatography (UHPLC) system (Agilent 1200, USA) for quantitative analysis of nucleotides [18,19].

### 2.8. Analysis of Volatile Flavor Compounds and Geosmin

The analysis of volatile compounds and geosmin in muscle tissue was performed using solid-phase microextraction (SPME) coupled with gas chromatography–mass spectrometry (GC-MS). Specifically, 3.0 g of minced muscle tissue was homogenized with 7.0 mL of deionized water in a 20 mL headspace vial. The vial was incubated in a 60 °C water bath for 20 min with magnetic stirring. Volatile compounds were then extracted by exposing a 50/30 μm DVB/CAR/PDMS SPME fiber to the sample headspace for 40 min at 60 °C under continuous stirring. Following extraction, the fiber was immediately desorbed in the GC injector port (Agilent 6890N, USA) for 5 min at 250 °C. Separation was achieved using a DB-5MS capillary column (30 m × 0.25 mm × 0.25 μm) with helium as carrier gas. The mass spectrometer (Agilent 5975B, USA) was operated in electron impact (EI) mode at 70 eV, with scanning from *m*/*z* 35 to 350. Identification was performed by comparing mass spectra with the NIST library and authentic standards, and quantification was carried out using internal standard (2-methyl-3-heptanone) calibration. All analyses were performed in triplicate [20].

### 2.9. Statistical Analysis

All data were analyzed using SPSS 25.0 and expressed as the mean ± standard error of the mean (mean ± S.E.M). Data were subjected to normality (Shapiro–Wilk test) and homogeneity of variance (Levene’s test) assessments prior to one-way analysis of variance (ANOVA). Differences among salinity groups were assessed using Tukey’s test. Meanwhile, independent samples *t*-tests were used to compare differences between each salinity group and the freshwater control (D0S0), as well as between different time points within the same salinity group. *p* < 0.05 was considered significant, and *p* < 0.01 was considered highly significant.

## 3. Results

### 3.1. Muscle Nutritional Composition in C. auratus gibelio

**Figure 1:**
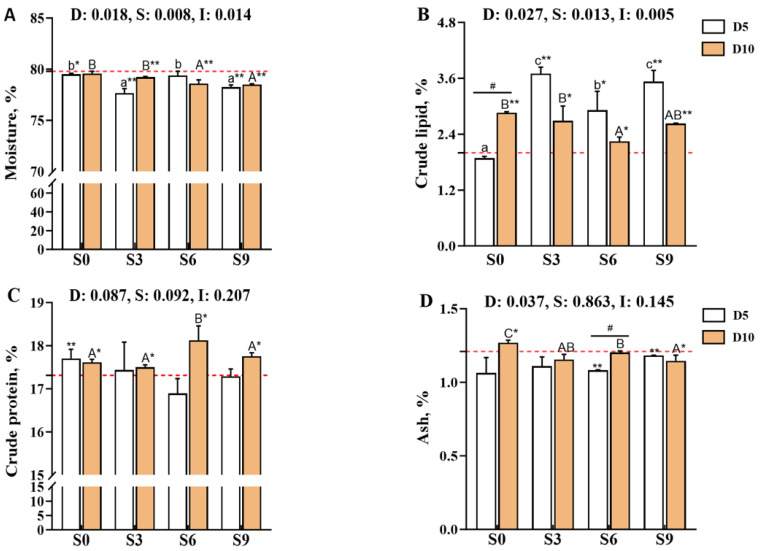
Effects of short-term depuration under saline conditions on muscle nutritional composition in *Carassius auratus gibelio*. (**A**), Moisture content in muscle; (**B**), Crude lipid content in muscle; (**C**), Crude protein content in muscle; (**D**), Ash content in muscle. The red dashed line indicates the baseline values of muscle nutritional composition measured before depuration (D0S0 group). “*” and “**” denote significant and highly significant differences between each depuration treatment group and the D0S0 group (independent samples *t*-test, *p* < 0.05 and *p* < 0.01, respectively). “#” denotes significant differences among groups subjected to different depuration durations under the same salinity conditions (Independent samples *t*-test, *p* < 0.05 and *p* < 0.01, respectively). Different lowercase superscript letters indicate significant differences among salinity groups (S0, S3, S6, and S9) after 5 days of depuration (D5) (Tukey’s test, *p* < 0.05), while different uppercase superscript letters indicate significant differences among salinity groups (S0, S3, S6, S9) after 10 days of depuration (D10) (Tukey’s test, *p* < 0.05).

The muscle moisture and crude lipid contents of *Carassius auratus gibelio* were significantly affected by the main effects of depuration duration, salinity level, and their interaction (*p* < 0.05; Figure 1A,B). Comparison with the pre-depuration group (D0S0) showed that all depuration treatments reduced muscle moisture content, and significant decreases were observed in the D5S0, D5S3, D5S9, D10S3, D10S6, and D10S9 groups (*p* < 0.05). Notably, after 10 days of depuration, the moisture content in the S6 and S9 groups was significantly lower than that in the S0 and S3 groups (*p* < 0.05). In contrast, depuration under saline conditions significantly increased the crude lipid content of muscle (*p* < 0.05 versus D0S0). After 5 days of depuration, the crude lipid content in the D5S3, D5S6, and D5S9 groups was significantly higher than that in the D5S0 group (*p* < 0.05), with the highest levels detected in the D5S3 and D5S9 groups (*p* < 0.05). After 10 days of depuration, the crude lipid content of muscle tended to decrease with an increase in salinity, and the D10S6 group exhibited significantly lower crude lipid levels than the D10S0 and D10S3 groups (*p* < 0.05).

The crude protein content of muscle was not significantly affected by the depuration duration, salinity level, or their interaction (*p* > 0.05, Figure 1C), whereas the ash content was influenced only by the main effect of depuration duration (*p* < 0.05; Figure 1D). Notably, 10 days of depuration under saline conditions significantly increased muscle crude protein content (*p* < 0.05 versus D0S0), with the D10S6 group showing significantly higher protein levels than the D10S0, D10S3, and D10S9 groups (*p* < 0.05). Comparisons with D0S0 showed that salinity levels of 3‰, 6‰, and 9‰ decreased the muscle ash content, with significant reductions observed in the D5S6, D5S9, and D10S9 groups (*p* < 0.05); among these, the D5S6 group exhibited significantly lower ash content than the D10S6 group (*p* < 0.05). After 10 days of depuration, the muscle ash content decreased with increasing salinity, and the D10S9 group showed significantly lower ash content than the D10S0 and D10S6 groups (*p* < 0.05).

### 3.2. Muscle Fatty Acid Composition and Nutritional Value in C. auratus gibelio

**Figure 2:**
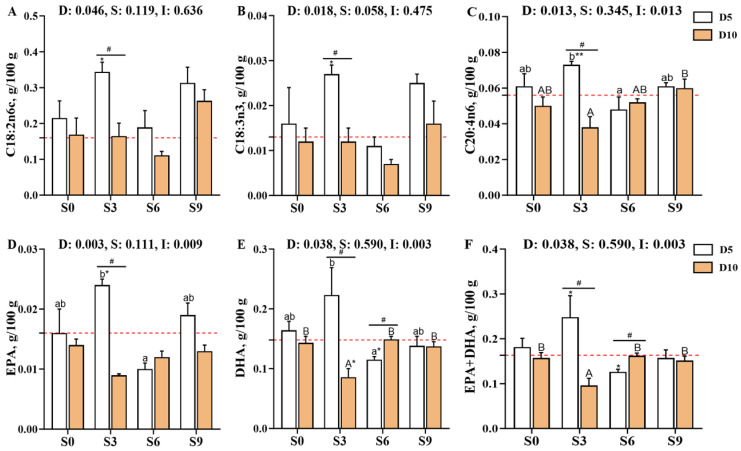
Effects of short-term depuration under saline conditions on muscle fatty acid composition in *Carassius auratus gibelio*. (**A**), C18:2n6c content in muscle; (**B**), C18:3n3 content in muscle; (**C**), C20:4n6 content in muscle; (**D**), C20:5n3 (EPA) content in muscle; (**E**), C22:6n3 (DHA) content in muscle; (**F**), Sum content of EPA+DHA in muscle. The red dashed line indicates the baseline values of muscle nutritional composition measured before depuration (D0S0 group). “*” and “**” denote significant and highly significant differences between each depuration treatment group and the D0S0 group (independent samples *t*-test, *p* < 0.05 and *p* < 0.01, respectively). “#” denotes significant differences among groups subjected to different depuration durations under the same salinity conditions (Independent samples *t*-test, *p* < 0.05). Different lowercase superscript letters indicate significant differences among salinity groups (S0, S3, S6, and S9) after 5 days of depuration (D5) (Tukey’s test, *p* < 0.05), while different uppercase superscript letters indicate significant differences among salinity groups (S0, S3, S6, S9) after 10 days of depuration (D10) (Tukey’s test, *p* < 0.05).

The concentrations of individual fatty acids in *Carassius auratus gibelio* muscle (C14:0, C16:0, C18:0, C16:1, C18:1n9c, C20:1, C22:1n9, C24:1, C18:2n6c [LA], C18:3n3 [ALA], C20:2, C20:3n6, C20:4n6 [ARA], C20:5n3 [EPA], and C22:6n3 [DHA]) were significantly affected by the main effect of depuration duration (*p* < 0.05, Figure 2, Supplementary Table S1). Among these, the contents of C20:1 and C20:2 were further influenced by salinity (*p* < 0.05), while those of C20:4n6 (ARA), C20:5n3 (EPA), and C22:6n3 (DHA) were affected by the interaction between depuration duration and salinity (*p* < 0.05). Compared with the pre-depuration group (D0S0), the D5S3 group showed significantly elevated contents of LA, ALA, ARA, EPA, DHA, and EPA + DHA (*p* < 0.05), and these values were also significantly higher than those in the D10S3 group (*p* < 0.05). After 10 days of depuration, the DHA and EPA + DHA contents in the S0, S6, and S9 groups were significantly higher than those in the S3 group (*p* < 0.05).

**Figure 3:**
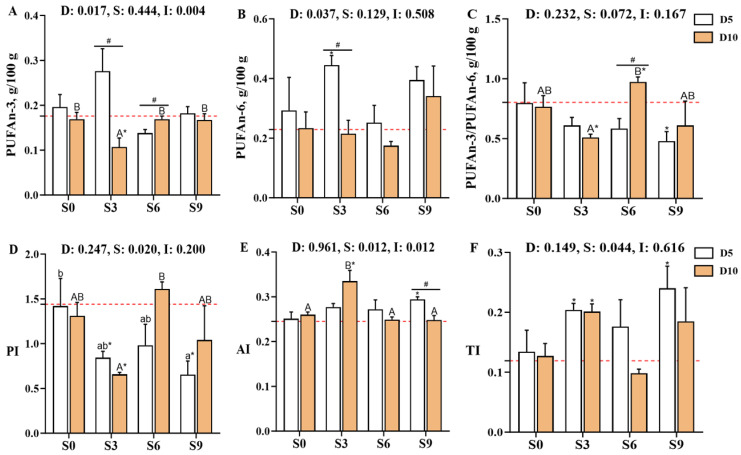
Effects of short-term depuration under saline conditions on muscle fatty acid nutritional evaluation in *Carassius auratus gibelio*. (**A**), Total content of n-3 polyunsaturated fatty acids (PUFAn-3) in muscle; (**B**), Total content of n-6 polyunsaturated fatty acids (PUFAn-6) in muscle; (**C**), The PUFAn-3/PUFAn-6 ratio in muscle; (**D**), Peroxidizability index (PI) in muscle; (**E**), Atherogenic index (AI) in muscle; (**F**), Thrombogenic index (TI) in muscle. The red dashed line indicates the baseline values of muscle nutritional composition measured before depuration (D0S0 group). “*” denote significant differences between each depuration treatment group and the D0S0 group (independent samples *t*-test, *p* < 0.05). “#” denotes significant differences among groups subjected to different depuration durations under the same salinity conditions (Independent samples *t*-test, *p* < 0.05). Different lowercase superscript letters indicate significant differences among salinity groups (S0, S3, S6, and S9) after 5 days of depuration (D5) (Tukey’s test, *p* < 0.05), while different uppercase superscript letters indicate significant differences among salinity groups (S0, S3, S6, S9) after 10 days of depuration (D10) (Tukey’s test, *p* < 0.05).

From a nutritional perspective, PUFA and PUFAn-6 levels were significantly influenced by depuration duration (*p* < 0.05, Figure 3, Supplementary Table S1), while PUFAn-3 levels were affected by both the depuration duration and its interaction with salinity (*p* < 0.05). The PI and TI indices were significantly affected by salinity (*p* < 0.05), whereas the AI was affected by both salinity and the interaction between salinity and depuration duration (*p* < 0.05). Notably, PUFA and PUFAn-6 levels were significantly higher in the D5S3 group than in the D0S0 (*p* < 0.05) and D10S3 (*p* < 0.05) groups. In contrast, PUFAn-3 levels were significantly decreased in the D10S3 group, which was accompanied by a significant increase in the n3/n6 and PI ratios (*p* < 0.05). The AI was also significantly elevated in D5S3, D5S9, and D10S3 groups (*p* < 0.05). Notably, after 10 days of depuration, muscle PUFAn-3 levels were significantly higher while AI values were significantly lower in the S0, S6, and S9 groups than in the S3 group, with the S6 group also showing significantly elevated n3/n6 and PI ratios (*p* < 0.05).

### 3.3. Muscle Amino Acid Composition and Nutritional Value Scores in C. auratus gibelio

**Figure 4:**
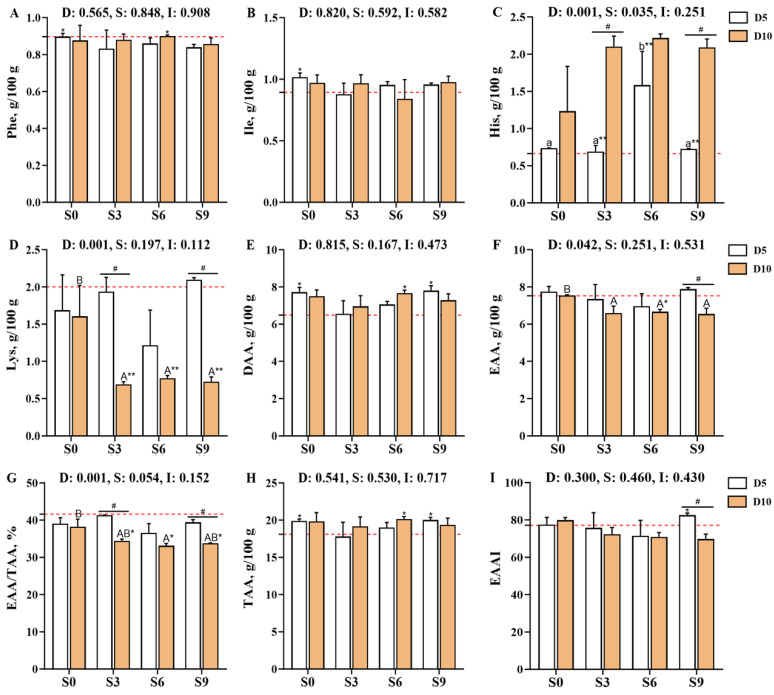
Effects of short-term depuration under saline conditions on muscle amino acid composition and essential amino acids in *Carassius auratus gibelio*. (**A**), Phenylalanine (Phe) content in muscle; (**B**), Isoleucine (Ile) content in muscle; (**C**), Histidine (His) content in muscle; (**D**), Lysine (Lys) content in muscle; (**E**), Total content of delicious amino acids (DAA) in muscle; (**F**), Total content of essential amino acids (EAA) in muscle; (**G**), The EAA/TAA ratio in muscle; (**H**), Total content of amino acid (TAA) in muscle; (**I**), Essential amino acid index (EAAI) in muscle. The red dashed line indicates the baseline values of muscle nutritional composition measured before depuration (D0S0 group). “*” and “**” denote significant and highly significant differences between each depuration treatment group and the D0S0 group (independent samples *t*-test, *p* < 0.05 and *p* < 0.01, respectively). “#” denotes significant differences among groups subjected to different depuration durations under the same salinity conditions (Independent samples *t*-test, *p* < 0.05). Different lowercase superscript letters indicate significant differences among salinity groups (S0, S3, S6, and S9) after 5 days of depuration (D5) (Tukey’s test, *p* < 0.05), while different uppercase superscript letters indicate significant differences among salinity groups (S0, S3, S6, S9) after 10 days of depuration (D10) (Tukey’s test, *p* < 0.05).

The contents of lysine (Lys), cysteine (Cys), and essential amino acids (EAAs) in *Carassius auratus gibelio* muscle, as well as the EAA/total amino acid (TAA) ratio, were significantly affected by the main effect of depuration duration. Meanwhile, histidine (His) levels were influenced by both the depuration duration and its interaction with salinity (*p* < 0.05, Figure 4, Supplementary Table S2). Among the EAAs, isoleucine (Ile) and phenylalanine (Phe) showed significantly higher levels in the D5S0 group and the D5S0 and D10S6 groups, respectively, compared with the D0S0 group. In contrast, the Lys content in the D10S3, D10S6, and D10S9 groups was significantly lower than that in the D0S0 group as well as the D5S3, D5S6, and D5S9 groups (*p* < 0.05).

With regard to umami amino acids, depuration treatment was found to increase the content of aspartate (Asp) in crucian carp muscle. Asp levels were significantly elevated in the D5S0, D5S3, D5S9, D10S0, and D10S6 groups when compared with the D0S0 group, while DAA levels were significantly higher in the D5S0, D5S9, and D10S6 groups (*p* < 0.05). After 10 days of depuration, the S3, S6, and S9 treatments increased the TAA content but decreased the EAA content and the EAA/TAA ratio. Notably, the TAA levels in the D10S6 group were significantly higher than those in the D0S0 group, whereas the EAA content and the EAA/TAA ratio were significantly lower (*p* < 0.05).

In addition, after 10 days of depuration, the Lys content in the D10S3, D10S6, and D10S9 groups was significantly lower than that in the D0S0 and D10S0 groups (*p* < 0.05). Correspondingly, the AAS and CS for Lys were significantly reduced in these groups when compared with the D0S0 group (*p* < 0.01, Supplementary Table S3). In contrast, the CS of Ile in the D5S0 group was significantly higher than that in the D0S0 group (*p* < 0.05). Notably, the D5S9 group exhibited a significantly higher EAAI than the D0S0 and D10S9 groups (*p* < 0.05). The EAAI was the highest in the D5S9 group, followed by the D10S0, D5S0, D0S0, D5S3, D10S3, D5S6, D10S6, and D10S9 groups. Based on AAS calculations, Met-Cys were identified as the limiting amino acids in the D0S0, D5S0, D5S3, D5S6, D5S9, and D10S0 groups. In contrast, Lys emerged as the main limiting amino acid in the D10S3, D10S6, and D10S9 groups, followed by Met-Cys. CS evaluations consistently indicated that Met-Cys were the main limiting amino acids across all groups, both before and after depuration.

### 3.4. Muscle Umami Nucleotides and Geosmin Content in C. auratus gibelio

**Figure 5:**
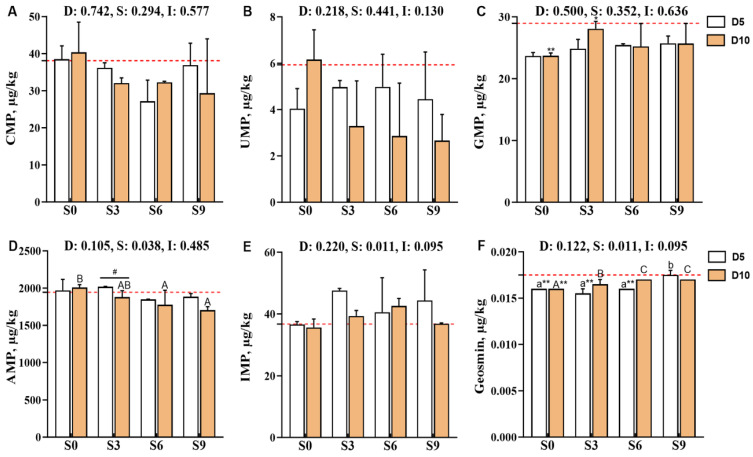
Effects of short-term depuration under saline conditions on the muscle content of flavor-related nucleotides and geosmin in *Carassius auratus gibelio*. (**A**), Cytidine monophosphate (CMP) content in muscle; (**B**), Uridine monophosphate (UMP) content in muscle; (**C**), Guanosine monophosphate (GMP) content in muscle; (**D**), Adenosine monophosphate (AMP) content in muscle; (**E**), Inosine monophosphate (IMP) content in muscle; (**F**), Geosmin content in muscle. The red dashed line indicates the baseline values of muscle nutritional composition measured before depuration (D0S0 group). “*” and “**” denote significant and highly significant differences between each depuration treatment group and the D0S0 group (independent samples *t*-test, *p* < 0.05 and *p* < 0.01, respectively). “#” denotes significant differences among groups subjected to different depuration durations under the same salinity conditions (Independent samples *t*-test, *p* < 0.05). Different lowercase superscript letters indicate significant differences among salinity groups (S0, S3, S6, and S9) after 5 days of depuration (D5) (Tukey’s test, *p* < 0.05), while different uppercase superscript letters indicate significant differences among salinity groups (S0, S3, S6, S9) after 10 days of depuration (D10) (Tukey’s test, *p* < 0.05).

Compared with the pre-depuration group (D0S0), the muscle guanosine monophosphate (GMP) content was significantly reduced in the D5S3 and D10S0 groups (*p* < 0.05, Figure 5). Specifically, the muscle adenosine monophosphate (AMP) content was significantly affected by the main effect of salinity level (*p* < 0.05). After 10 days of depuration, AMP levels decreased with increasing salinity levels, with the D10S6 and D10S9 groups showing significantly lower AMP levels than the D10S0 group (*p* < 0.05). Moreover, the AMP levels in the D10S3 group were significantly lower than those in the D5S3 group (*p* < 0.05). Additionally, the geosmin content of muscle tissues was also significantly affected by the salinity level (*p* < 0.05). Notably, depuration treatments consistently reduced geosmin contents, with the D5S0, D5S3, D5S6, and D10S0 groups showing extremely significant reductions compared with the D0S0 group (*p* < 0.01). However, geosmin showed gradual accumulation in muscle tissues as the salinity level increased, and its content was significantly higher in the S9 group than in the S0 and S3 groups (*p* < 0.05).

### 3.5. Muscle Volatile Compound Contents in C. auratus gibelio

**Figure 6:**
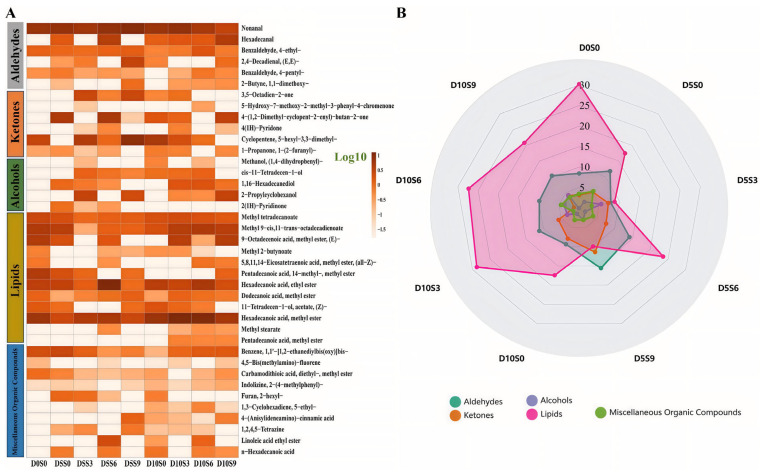
Effects of short-term depuration under saline conditions on the muscle content of volatile compounds in *Carassius auratus gibelio*. (**A**) the heatmap analysis of volatile compounds. The color gradient from light to dark represents the relative abundance of volatile compounds on a Log_10_ scale, with darker colors indicating higher concentrations. (**B**) the classification analysis of volatile compounds.

A total of 39 volatile compounds were detected in the muscle, including 6 aldehydes, 6 ketones, 5 alcohols, 12 lipids, and 10 other compounds (Figure 6A, Supplementary Table S4). There are relatively higher levels of lipids, aldehydes, and ketones in *C. auratus gibelio* muscle (Figure 5B). Compared with the pre-depuration group (D0S0), the aldehyde contents increased in the muscle after 5 days or 10 days of depuration, while except in the D5S0 group. The alcohols contents were higher in the D5S0, D5S3, D5S6, D5S9, D10S0, D10S3, D10S6, and D10S9 groups than in the D0S0 group, whereas the lipids contents were lower. Compared with the pre-depuration group (D0S0), the hexanal contents increased in the muscle, with 4-ethylbenzaldehyde elevated in the D10S6 group, methyl laurate and 1,2-diphenoxyhexane increased in the D5S3 group. After 5 days of purification, the S3 and S9 treatments increased 5-hexyl-3,3-dimethylcyclopentene contents in the muscle. After 10 days of purification, the S6 treatment increased the contents of methyl myristate and ethyl palmitate in the muscle.

## 4. Discussion

Water salinity is a key determinant of fish muscle quality. Under conditions of salt stress, fish must produce nutrients to drive osmoregulatory processes and maintain osmotic homeostasis [21]. Studies have shown that *Scophthalmus maximus* typically allocate 10–20% of their total energy budget towards sustaining osmotic balance, and the magnitude of energy expenditure is closely related to species-specific traits, ecological habits, as well as salinity concentration and the duration of depuration treatment. Such physiological responses alter the body composition of fish and consequently affect muscle quality [22]. In the present study, depuration under saline conditions was found to decrease the moisture and ash contents of fish while increasing the crude protein and crude lipid contents. Similar findings have been reported previously. For example, *Lateolabrax japonicus* reared in seawater were found to exhibit reduced moisture and ash contents as well as elevated crude protein levels [23]. In the early stage of freshwater depuration, murray cod (*Maccullochella peelii*) and rainbow smelt (*Osmerus mordax Mitchell)* were also found to show comparable trends. These phenomena could be attributed to the reduction of water retention in freshwater fish via fluid regulation under salt stress, as well as the simultaneous enhancement of protein synthesis to maintain osmotic balance [24,25]. Moreover, studies have also shown that fish can preferentially mobilize lipid reserves from the liver instead of muscle lipids under conditions of salt stress [25]. Under stable environmental conditions, carbohydrates serve as an adequate substrate to meet the daily energy requirements of fish [26]. When fish are subjected to environmental stress, they tend to consume more energy during the adaptation process, at which point proteins and lipids become important energy sources [27]. In grass carp (*Ctenopharyngodon idella*), the expression levels of lipogenesis-related genes fatty acid synthase (*fas*) and acetyl-CoA carboxylase alpha (*acca*) in muscle tissue increased with rising salinity, while the expression of the lipid metabolism gene lipoprotein lipase (*lpl*) showed a similar upward trend, reaching significantly higher levels. Variations in the intensity of environmental stress experienced by fish can lead to differential metabolic responses, ultimately influencing the composition of muscle body components [28].

Fatty acid composition is an important indicator for evaluating muscle quality and nutritional value [29]. Oleic acid and linoleic acid, which are essential nutrients for humans, not only reduce the serum levels of harmful cholesterol (e.g., LDL) but also help maintain the levels of beneficial cholesterol (e.g., HDL), thereby helping in the prevention of cardiovascular and cerebrovascular diseases [30]. The PUFAs EPA and DHA are also indispensable for human growth and development, playing a vital role in enhancing memory and brain function, as well as supporting visual and cognitive development in infants and young children [31,32]. In the present study, the muscle PUFA content in the D5S3 group was found to be significantly elevated, accompanied by a notable increase in functional unsaturated fatty acids such as oleic acid, linoleic acid, and the total amount of EPA + DHA. Previous studies have demonstrated that the PUFA levels in fish are affected by environmental salinity [33]. For instance, *Oncorhynchus mykiss* reared in seawater exhibit higher muscle PUFA contents than those cultured in freshwater [34], while *Lateolabrax japonicus* reared in seawater show significantly higher oleic acid and linoleic acid levels [23]. Notably, salinity treatments have also been confirmed to promote the deposition of EPA and DHA in muscle across various fish species [35,36]. The n-3/n-6 PUFA ratio in fish muscle, which has important nutritional and health implications, generally ranges from 0.24 to 4.1 [37]. Notably, a higher n-3/n-6 ratio promotes the regulation of lipid metabolism and suppresses inflammatory responses [38]. In the present study, the increase in salinity and depuration duration (10 days at 6‰) was found to significantly enhance the n-3/n-6 ratio. Similarly, a study on *Ctenopharyngodon idella* reported that depuration at 7.5‰ salinity led to significantly higher levels of n-3 PUFAs, EPA, DHA, and total PUFAs in fish muscle compared with freshwater rearing [10]. Fatty acid desaturase 2 (*fads2*) and elongase of very long-chain fatty acids (*elovl*) are two key rate-limiting enzymes in the LC-PUFA biosynthetic pathway of fish, whose synergistic interaction directly determines the composition and content of polyunsaturated fatty acids in fish. In *Oncorhynchus mykiss*, elovl4b1 and elovl4b2 can convert 20:5n-3 and 22:5n-3 to 24:5n-3 [39].

Compared with other nutritional indicators, the AI and TI of fatty acids more comprehensively reflect the potential risk of atherosclerosis and thrombosis. The lower the values, the better the fatty acid composition and the lower the risk of coronary heart disease [37]. Previously, *Oncorhynchus mykiss* reared in 6‰ seawater have shown reduced muscle AI and TI values [40]. In the present study, the AI in all depuration groups ranged from 0.24 to 0.29, while the TI ranged from 0.09 to 0.24. Importantly, both these values were lower than the reported ranges for other fish species (AI: 0.33–2.37; TI: 0.01–1.18) [41,42]. Additionally, both the AI and TI of muscle fatty acids reached their lowest levels in *C. auratus gibelio* after 10 days of depuration under 6‰ salinity.

The composition of hydrolyzed amino acids is an important indicator of the nutritional value of fish protein. Moreover, umami amino acids, such as the strongly savory Asp and glutamate (Glu), greatly influence fish flavor [43]. In this study, depuration treatments increased the contents of Asp and total umami amino acids to varying degrees, with significant changes observed in the D5S0, D5S9, and D10S6 groups. Previous reports have indicated that depuration under saline conditions can enhance the content of umami amino acids in fish muscle. For example, grass carp reared under 6‰ salinity showed significantly higher levels of umami amino acids than freshwater-reared fish [11]. In the present study, the TAA content in the D5S0, D5S9, and D10S6 groups was found to be significantly elevated, reflecting an overall improvement in the nutritional value of muscle amino acids [44]. However, when depuration was extended to 10 days, the increase in salinity led to a reduction in muscle Lys content, as well as a reduction in the AAS and CS of Lys. Internationally, the AAS, CS, and EAAI are widely used as indicators for assessing the nutritional value of EAAs. Values closer to 1 for the AAS and CS, or closer to 100 for the EAAI, reflect higher protein quality and a more balanced amino acid profile [45]. For instance, after micro-flow starvation treatment, the hybrid crucian carp “Pioneer 1” was found to exhibit AAS and CS values closer to 1 and EAAI values closer to 100 [46]. In the present study, Lys was identified as the primary limiting amino acid in fish muscle after 10 days of depuration at 3‰, 6‰, and 9‰ salinity. This could be due to the higher Lys requirement of hybrid crucian carp in saline water [47], consistent with previous findings from tilapia, wherein juvenile fish reared at 8‰ salinity exhibited higher Lys demands than those reared in freshwater [48]. Therefore, our findings indicated that when employing salinity-based depuration for freshwater fish, excessively long depuration periods that could lead to reduced muscle Lys contents should be avoided.

Geosmin is one of the primary sources of the earthy odor in fish [49]. The gills serve as the main site of geosmin absorption in fish, and when fish are transferred to clean water, geosmin can be eliminated from the body via diffusion. This physiological process forms the basis of geosmin removal through depuration in aquaculture [50]. In the present study, salinity-based depuration effectively reduced geosmin accumulation in muscle to varying degrees, with significant decreases observed after 5 days of depuration under salinity levels of 0‰, 3‰, and 6‰. Previous studies have reported that the lipophilic nature of geosmin facilitates its accumulation in fatty tissues [51], suggesting that the positive correlation observed between water salinity and muscle geosmin content in this study may be related to the concomitant increase in the crude lipid content.

Free nucleotides such as AMP, GMP, and IMP act as important contributors to the umami taste of seafood products. These nucleotides not only impart a savory flavor themselves but also act synergistically with umami amino acids (Asp and Glu) to enhance fish flavor [52]. Cells undergoing energy imbalance can lead to excessive activation of AMP deaminase, resulting in accelerated depletion of adenine nucleotides. IMP is primarily derived from ATP degradation in muscle tissues, and under conditions of high energy consumption, AMP can be converted into IMP to conserve AIP and maintain energy homeostasis [53,54]. In this study, the IMP content tended to increase during depuration under saline conditions, and this was accompanied by a decrease in AMP levels. These dynamics likely reflected the increased energy expenditure in *C. auratus gibelio*, which was required to maintain physiological functions under saline conditions. The findings were also consistent with previous observations of elevated IMP levels in the muscle of seawater-reared *Micropterus salmoides* [13].

Finally, aldehydes are the main products of lipid oxidative degradation and amino acid Strecker synthesis. Branched short-chain aldehydes and unsaturated aldehydes are generated from amino acid deamination and fatty acid degradation, respectively, and are characterized by pleasant floral, fruity, nutty, and sweet aromas. Due to their low odor thresholds, aldehydes are considered key contributors to the characteristic aroma of aquatic products [55]. Hexanal, which imparts a grassy flavor, is an important indicator for evaluating the oxidative state and flavor quality of meat, and can be generated through the oxidative degradation of C20:4n6 [56,57]. In the present study, hexanal levels were higher in all salinity-treated groups, consistent with the increase in C20:4n6 content in the muscle induced by salinity purification. Ester compounds, formed through lipid oxidation in muscle tissues, generally produce fruity, buttery, and fatty aromas [58]. The dynamic balance of lipid metabolism plays a crucial regulatory role in the flavor formation of aquatic products. Studies have demonstrated that CD36 overexpression-induced lipid overload promotes the cellular uptake of long-chain fatty acids (LCFAs), leading to the storage of excess fatty acids as triglycerides (TG) and their subsequent conversion into more metabolically active lipid metabolites such as diacylglycerols (DG) and ceramides (CER). This lipid remodeling process is closely associated with the generation of flavor precursors. In addition to lipogenesis, catabolic processes including fatty acid oxidation (FAO) and lipolysis also play key roles in maintaining cellular lipid homeostasis. Esters produced during lipid oxidation, particularly short-chain esters generated via the β-oxidation pathway, impart characteristic aromas such as fruity, buttery, and fatty notes to muscle tissues [59]. The present results showed that the contents of methyl laurate, which has a honey-like aroma, and 1,2-diphenoxyethane, which presents an aromatic odor, were elevated in the D5S3 group. Most ketone compounds possess characteristic fruity and creamy aromas [60] and play an important role in masking the fishy odor of aquatic products. In this study, the contents of ketone compounds were generally increased in all salinity-treated groups, likely due to the activation of energy metabolism pathways induced by salinity stress, as these ketones serve as intermediates in energy metabolism [61]. Consequently, salinity purification enhanced energy metabolism and ketone production, and the coordinated changes in volatile compounds collectively promoted the accumulation of pleasant aromatic substances in *C. auratus gibelio* muscle.

## 5. Conclusions

This study demonstrates that short-term depuration under saline conditions can improve the muscle nutritional composition and quality of *C. auratus gibelio*. Rearing under 6‰ salinity for 10 days significantly enhanced the contents of crude protein, total amino acids, and total umami amino acids in muscle tissues, while also improving the muscle fatty acid composition and related cardiovascular benefits. Depuration under 9‰ salinity for 5 days significantly increased the content of the functional PUFAs, EPA, and DHA, and improved the EAAI. However, the findings indicated that for short-term cultivation under high salinity, attention should be paid to the potential accumulation of geosmin in muscle and the supplementation of limiting amino acids. Future research should optimize salinity conditions to maximize nutritional gains while minimizing geosmin accumulation and amino acid imbalances.

## Data Availability

The original contributions presented in this study are included in the article/Supplementary Materials. Further inquiries can be directed to the corresponding author.

## Supplementary Materials

The following supporting information can be downloaded at: https://www.mdpi.com/article/10.3390/foods14234155/s1, Table S1. Effects of short-term depuration under saline conditions on muscle fatty acid composition and health assessment indices in *Carassius auratus gibelio*. Table S2. Effects of short-term depuration under saline conditions on muscle amino acid composition and content in *Carassius auratus gibelio*. Table S3. Effects of short-Term depuration under saline conditions on muscle essential amino acid score in *Carassius auratus gibelio*. Table S4. Effects of short-term depuration under saline conditions on major volatile compounds in muscle of *Carassius auratus gibelio*.
