# Supplementary material for: Effects of Short-Term Depuration on Muscle Nutritional Quality and Flavor Composition in Carassius auratus gibelio
Source: Foods. 2025 Dec 3;14(23):4155. doi: 10.3390/foods14234155 (PMC12692719; doi:10.3390/foods14234155)
Supplement: Supplementary file 1 [file foods-14-04155-s001.zip › foods-3956276-supplementary.pdf]

Supplementary Tables

Table S1. Effects of short-term depuration under saline conditions on muscle fatty acid composition and health assessment indices in *Carassius auratus gibelio.*

**g/100g**	**D0S0**	**D5**				**D10**				**Two-way ANOVA (*P* value)**		
		**S0**	**S3**	**S6**	**S9**	**S0**	**S3**	**S6**	**S9**	**D**	**S**	**D×S**
C14:0	0.009±0.003	0.013±0.007	0.026±0.002*	0.012±0.004	0.025±0.003*	0.010±0.003	0.014±0.003$^{\#}$	0.006±0.001	0.015±0.006	**0.026**	0.056	0.750
C16:0	0.117±0.017	0.150±0.053	0.294±0.052*	0.153±0.052	0.259±0.042*	0.126±0.026	0.145±0.023	0.101±0.008	0.188±0.059	**0.027**	0.069	0.519
C18:0	0.059±0.003	0.063±0.009	0.091±0.009*	0.065±0.011	0.084±0.005*	0.055±0.004	0.062±0.005	0.054±0.001	0.075±0.016	**0.042**	0.067	0.612
SFA	0.185±0.023	0.227±0.070	0.227±0.064	0.227±0.067	0.227±0.051	0.192±0.034	0.192±0.032	0.192±0.011	0.192±0.081	**0.028**	0.066	0.558
C16:1	0.018±0.006	0.026±0.015	0.095±0.034	0.030±0.016	0.075±0.016*	0.020±0.006	0.027±0.006	0.011±0.002	0.035±0.015	**0.014**	0.058	0.31
C18:1n9c	0.159±0.041	0.231±0.115	0.568±0.098*	0.262±0.116	0.517±0.130	0.186±0.058	0.226±0.049$^{\#}$	0.114±0.014	0.381±0.172	**0.039**	0.057	0.567
C20:1	0.014±0.005	0.017±0.008^{a}	0.046±0.008^{b*}	0.020±0.005^{a}	0.033±0.004^{ab*}	0.015±0.003	0.018±0.003$^{\#}$	0.009±0.001	0.031±0.012	**0.045**	**0.029**	0.247
C22:1n9	0.020±0.006	0.011±0.001	0.013±0.001	0.010±0.001	0.010±0.001	0.012±0.001	0.014±0.001	0.016±0.004	0.015±0.001	**0.038**	0.854	0.438
C24:1	0.005±0.000	0.004±0.001	0.005±0.001	0.005±0.001	0.004±0.001	0.004±0.001	0.004±0.001	0.004±0.001	0.005±0.001	**0.045**	0.322	0.182
MUFA	0.215±0.047	0.291±0.139	0.728±0.139*	0.328±0.139	0.640±0.149	0.239±0.070	0.290±0.060$^{\#}$	0.155±0.016	0.467±0.200	**0.034**	0.051	0.502
C18:2n6c	0.160±0.039	0.215±0.098	0.344±0.027*	0.189±0.047	0.313±0.044	0.169±0.046	0.165±0.036$^{\#}$	0.111±0.011	0.263±0.091	**0.046**	0.119	0.636
C18:3n3	0.013±0.004	0.016±0.008	0.027±0.002*	0.011±0.002	0.025±0.002	0.012±0.003	0.012±0.003$^{\#}$	0.007±0.001	0.016±0.005	**0.018**	0.058	0.475
C20:2	0.008±0.002	0.008±0.003^{ab}	0.017±0.001^{b*}	0.008±0.001^{a}	0.013±0.001^{ab*}	0.007±0.001	0.008±0.001$^{\#}$	0.006±0.001	0.012±0.003	**0.032**	**0.016**	0.116
C20:3n6	0.012±0.002	0.016±0.005^{ab}	0.026±0.002^{b*}	0.014±0.003^{a}	0.021±0.001^{ab*}	0.014±0.002	0.011±0.002$^{\#}$	0.010±0.001	0.017±0.004	**0.023**	0.172	0.203

g/100g	D0S0	D5				D10				Two-way ANOVA (*P* value)		
		S0	S3	S6	S9	S0	S3	S6	S9	D	S	D×S
C20:4n6	0.056±0.001	0.061±0.007^{ab}	0.073±0.002^{b**}	0.048±0.007^{a}	0.061±0.002^{ab}	0.050±0.005^{AB}	0.038±0.006$^{A\#}$	0.052±0.002^{AB}	0.060±0.005^{B}	**0.013**	0.345	**0.013**
C20:5n3(EPA)	0.016±0.002	0.016±0.004^{ab}	0.024±0.001^{b*}	0.010±0.001^{a}	0.019±0.002^{ab}	0.014±0.001	0.009±0.002$^{\#}$	0.012±0.001	0.013±0.001	**0.003**	0.111	**0.009**
C22:6n3(DHA)	0.148±0.007	0.164±0.015^{ab}	0.223±0.046^{b}	0.115±0.005^{a*}	0.138±0.016^{ab}	0.143±0.011^{B}	0.086±0.014$^{A*\#}$	0.149±0.005$^{B\#}$	0.137±0.008^{B}	**0.038**	0.590	**0.003**
PUFA	0.413±0.055	0.499±0.142^{ab}	0.738±0.084^{b*}	0.398±0.067^{a}	0.591±0.034^{ab}	0.411±0.071	0.330±0.066$^{\#}$	0.351±0.022	0.521±0.115	**0.020**	0.167	0.147
C20:5n3+C22:6n3	0.163±0.034	0.181±0.020	0.248±0.048*	0.126±0.006*	0.157±0.018	0.157±0.012^{B}	0.096±0.016$^{A\#}$	0.162±0.006$^{B\#}$	0.151±0.010^{B}	**0.026**	0.538	**0.003**
PUFAn-3	0.176±0.012	0.197±0.028	0.276±0.050	0.138±0.008	0.182±0.015	0.169±0.015^{B}	0.107±0.020$^{A*\#}$	0.169±0.007$^{B\#}$	0.167±0.014^{B}	**0.017**	0.444	**0.004**
PUFAn-6	0.229±0.041	0.293±0.111	0.445±0.032*	0.252±0.058	0.395±0.045	0.234±0.054	0.215±0.045$^{\#}$	0.175±0.014	0.341±0.101	**0.037**	0.129	0.508
n3/n6	0.803±0.092	0.797±0.169	0.611±0.066	0.585±0.083	0.479±0.080*	0.766±0.093^{AB}	0.510±0.027^{A*}	0.974±0.041$^{B*\#}$	0.611±0.202^{AB}	0.232	0.072	0.167
UFA	0.628±0.102	0.791±0.281	1.467±0.221*	0.727±0.207	1.232±0.182*	0.650±0.141	0.621±0.127$^{\#}$	0.507±0.037	0.989±0.310	**0.024**	0.078	0.326
PI	1.440±0.167	1.420±0.308^{b}	0.842±0.071^{ab*}	0.981±0.235^{ab}	0.655±0.150^{a*}	1.310±0.150^{AB}	0.657±0.021^{A*}	1.610±0.079^{B}	1.040±0.382^{AB}	0.247	**0.020**	0.200
AI	0.245±0.012	0.251±0.015	0.277±0.008	0.272±0.021	0.294±0.006*	0.260±0.006^{A}	0.335±0.024^{B*}	0.249±0.006^{A}	0.248±0.010$^{A\#}$	0.961	**0.012**	**0.012**
TI	0.119±0.016	0.134±0.036	0.204±0.011*	0.176±0.045	0.240±0.037*	0.127±0.021	0.201±0.013*	0.098±0.007	0.185±0.056	0.149	**0.044**	0.616

Note: "*" and "**" denote significant and highly significant differences between each depuration treatment group and the D0S0 group (independent samples *t*-test, $P < 0.05$ and $P < 0.01$, respectively). "#" and "##" denote significant and highly significant differences among groups subjected to different depuration durations under the same salinity conditions (Independent samples *t*-test, $P < 0.05$ and $P < 0.01$, respectively). Different lowercase superscript letters indicate significant differences among salinity groups (S0, S3, S6, and S9) after 5 days of depuration (D5) (Tukey's test, $P < 0.05$), while different uppercase superscript letters indicate significant differences among salinity groups (S0, S3, S6, S9) after 10 days of depuration (D10) (Tukey's test, $P < 0.05$). Consistent with the table note below.

Table S2. Effects of short-term depuration under saline conditions on muscle amino acid composition and content in *Carassius auratus gibelio.*

g/100g	D0S0	D5				D10				Two-way ANOVA (*P* value)		
		S0	S3	S6	S9	S0	S3	S6	S9	D	S	D×S
Asp$^{\Omega\triangle}$	1.843±0.031	2.183±0.080*	1.883±0.159**	2.020±0.012	2.09±0.052*	2.090±0.078*	1.927±0.145	2.130±0.047**	1.933±0.084	0.720	0.131	0.492
Glu $^{\Omega\triangle}$	2.253±0.598	2.990±0.162	2.500±0.320	2.570±0.100	2.973±0.065	2.950±0.131	2.687±0.319	2.980±0.092	2.890±0.197	0.407	0.246	0.582
Gly$^{\Omega\triangle}$	1.050±0.121	1.100±0.025^{ab}	0.940±0.110^{a}	1.077±0.024^{ab}	1.267±0.133^{b}	1.063±0.048	1.007±0.047	1.117±0.023$^{\#}$	1.067±0.022	0.506	0.074	0.234
Ala$^{\Omega\triangle}$	1.337±0.107	1.437±0.037	1.230±0.137	1.390±0.031	1.467±0.069	1.390±0.100	1.330±0.067	1.437±0.045	1.387±0.052	0.926	0.206	0.616
Thr$^{\blacktriangle}$	0.880±0.050	0.983±0.028	0.857±0.090	0.917±0.033	0.927±0.009	0.947±0.052	0.913±0.062	0.973±0.022	0.920±0.050	0.625	0.438	0.723
Val$^{\blacktriangle}$	1.023±0.067	1.150±0.025	0.997±0.097	1.090±0.031	1.080±0.010	1.090±0.075	1.103±0.047	1.140±0.030	1.097±0.062	0.472	0.569	0.503
Met$^{\blacktriangle}$	0.397±0.084	0.340±0.110	0.383±0.068	0.360±0.110	0.427±0.022	0.453±0.071	0.473±0.018	0.380±0.084	0.380±0.017	0.401	0.882	0.691
Ile$^{\blacktriangle}$	0.893±0.049	1.017±0.033*	0.877±0.090	0.953±0.027	0.957±0.012	0.970±0.065	0.967±0.069	0.840±0.157	0.977±0.047	0.820	0.592	0.582
Leu$^{\blacktriangle}$	1.517±0.090	1.680±0.050	1.460±0.153	1.570±0.050	1.553±0.019	1.597±0.107	1.563±0.112	1.660±0.032	1.583±0.078	0.575	0.494	0.702
Lys$^{\blacktriangle}$	2.000±0.098	1.687±0.474	1.937±0.192	1.217±0.472	2.097±0.027	1.603±0.417^{B}	0.690±0.035$^{A**\#}$	0.773±0.035^{A**}	0.727±0.063$^{A**\#}$	**0.001**	0.197	0.112
Phe$^{\blacktriangle}$	0.813±0.035	0.897±0.018*	0.833±0.100	0.860±0.030	0.840±0.015	0.877±0.082	0.880±0.031	0.900±0.006*	0.857±0.033	0.565	0.848	0.908
Tyr$^{\triangle}$	0.583±0.042	0.617±0.037	0.580±0.070	0.560±0.057	0.573±0.015	0.610±0.055	0.613±0.037	0.570±0.056	0.557±0.019	0.882	0.669	0.954
Cys$^{\triangle}$	0.087±0.040	0.085±0.025	0.089±0.018	0.076±0.027	0.159±0.033	0.153±0.013	0.183±0.026$^{\#}$	0.160±0.030	0.120±0.015	**0.008**	0.742	0.051
Ser$^{\triangle}$	0.700±0.040	0.783±0.028	0.677±0.069	0.717±0.034	0.733±0.009	0.760±0.040	0.710±0.040	0.767±0.009	0.723±0.043	0.655	0.280	0.754
Pro$^{\triangle}$	0.74 0±0.087	0.753±0.038	0.633±0.081	0.717±0.023	0.76±0.045	0.687±0.042	0.707±0.049	0.733±0.027	0.713±0.044	0.862	0.513	0.449

g/100g	D0S0	D5				D10				Two-way ANOVA (*P* value)		
		**S0**	**S3**	**S6**	**S9**	**S0**	**S3**	**S6**	**S9**	**D**	**S**	**D×S**
His$^{\&}$	0.663±0.058	0.737±0.003^{a}	0.690±0.081^{a**}	1.583±0.454^{b**}	0.727±0.003^{a**}	1.233±0.603	2.103±0.140$^{\#}$	2.217±0.055	2.093±0.111$^{\#}$	**0.001**	**0.035**	0.251
Arg$^{\&}$	1.317±0.097	1.443±0.064	1.223±0.135	1.333±0.052	1.390±0.032	1.353±0.089	1.303±0.092	1.367±0.039	1.337±0.052	0.891	0.369	0.675
DAA	6.483±0.620	7.710±0.262*	6.553±0.705	7.057±0.163	7.797±0.261*	7.493±0.340	6.950±0.576	7.663±0.160*	7.277±0.347	0.815	0.167	0.473
EAA	7.523±0.307	7.753±0.269	7.343±0.786	6.967±0.664	7.880±0.082	7.537±0.038^{B}	6.590±0.372^{A}	6.667±0.122^{A*}	6.540±0.315$^{A\#}$	**0.042**	0.251	0.531
EAA/TAA	41.593±0.984	39.025±1.604	41.287±0.062	36.537±2.52	39.383±0.737	38.248±2.004^{B}	34.442±0.443$^{AB*\#}$	33.105±0.621^{A*}	33.776±0.057$^{AB*\#}$	**0.001**	0.054	0.152
NEAA	8.594±0.584	9.94±0.380	8.531±0.921	9.121±0.301	10.021±0.310	9.701±0.462	9.161±0.677	9.891±0.254*	9.391±0.423*	0.721	0.259	0.467
NEAA/TAA	47.47±0.679	50.012±1.384	47.966±0.016	48.017±0.140	50.041±0.751	49.008±0.731	47.781±0.475	49.11±0.545	48.506±0.211	0.403	0.103	0.265
CEAAs	1.980±0.145	2.18±0.070	1.912±0.210	2.921±0.432	2.121±0.032	2.591±0.690	3.402±0.232$^{\#}$	3.581±0.092*	3.433±0.163$^{\#}$	**0.001**	0.088	0.299
TAA	18.097±0.966	19.881±0.271*	17.789±1.919	19.009±0.668	20.019±0.341*	19.827±1.171	19.160±1.266	20.143±0.327*	19.360±0.900	0.541	0.530	0.717

Table S3. Effects of short-Term depuration under saline conditions on muscle essential amino acid score in *Carassius auratus gibelio.*

EAA evaluation		D0S0	D5				D10				Two-way ANOVA (*P* value)		
			S0	S3	S6	S9	S0	S3	S6	S9	D	S	D×S
AAS	IIe	1.06 ± 0.03	1.21 ± 0.04	1.04 ± 0.10	1.13 ± 0.03	1.14 ± 0.01	1.15 ± 0.07	1.15 ± 0.08	1.00 ± 0.18	1.16 ± 0.05	0.81	0.58	0.59
	Leu	1.02 ± 0.03	1.13 ± 0.03	0.98 ± 0.10	1.06 ± 0.03	1.05 ± 0.01	1.08 ± 0.07	1.05 ± 0.07	1.12 ± 0.02	1.07 ± 0.05	0.57	0.47	0.69
	Lys	1.75 ± 0.05	1.47 ± 0.41	1.69 ± 0.16	1.06 ± 0.41	1.83 ± 0.02	1.40 ± 0.36	0.60±0.03[**#]	0.68 ± 0.03[**]	0.63±0.05[**#]	**0.01**	0.20	0.11
	Thr	1.05 ± 0.03	1.17 ± 0.03	1.02 ± 0.10	1.09 ± 0.04	1.10 ± 0.01	1.12 ± 0.06	1.08 ± 0.07	1.16 ± 0.02	1.09 ± 0.05	0.61	0.43	0.71
	Val	0.98 ± 0.03	1.10 ± 0.02	0.95 ± 0.09	1.04 ± 0.02	1.04 ± 0.01	1.04 ± 0.07	1.05 ± 0.04	1.09 ± 0.02	1.05 ± 0.05	0.49	0.54	0.50
	Met-Cys	0.65 ± 0.09	0.57± 0.18	0.63± 0.09	0.59 ± 0.17	0.79 ± 0.02	0.82 ± 0.09	0.89 ± 0.01	0.73 ± 0.11	0.67 ± 0.04	0.12	0.81	0.34
	Phe-Tyr	1.09 ± 0.02	1.18 ± 0.04	1.10 ± 0.13	1.11 ± 0.06	1.10 ± 0.01	1.16 ± 0.10	1.17 ± 0.05	1.15 ± 0.04	1.10 ± 0.03	0.69	0.81	0.94
CS	IIe	0.80 ± 0.02	0.90± 0.03[*]	0.78 ± 0.08	0.85 ± 0.02	0.86 ± 0.01	0.87 ± 0.05	0.87 ± 0.06	0.75 ± 0.14	0.88 ± 0.04	0.85	0.58	0.58
	Leu	0.84 ± 0.02	0.93 ± 0.02	0.81 ± 0.08	0.87 ± 0.02	0.86 ± 0.01	0.89 ± 0.06	0.87 ± 0.06	0.92 ± 0.01	0.88 ± 0.04	0.57	0.55	0.72
	Lys	1.35 ± 0.03	1.14 ± 0.32	1.30 ± 0.12	0.82 ± 0.32	1.41 ± 0.01	1.08 ± 0.28	0.51±0.02[**#]	0.52 ±0.02[**]	0.49±0.04[**#]	**0.01**	0.20	0.11
	Thr	0.90 ± 0.02	1.00 ± 0.02	0.87 ± 0.09	0.93 ± 0.03	0.94 ± 0.01	0.96 ± 0.05	0.93 ± 0.06	0.99 ± 0.02	0.94 ± 0.05	0.61	0.43	0.72
	Val	0.74 ± 0.02	0.83 ± 0.01	0.72 ± 0.06	0.79 ± 0.02	0.78 ± 0.01	0.79 ± 0.05	0.80 ± 0.03	0.82 ± 0.02	0.79 ± 0.04	0.46	0.56	0.50
	Met-Cys	0.37 ± 0.05	0.33 ± 0.10	0.36 ± 0.05	0.33 ± 0.09	0.45 ± 0.01	0.46 ± 0.05	0.50 ± 0.01	0.41 ± 0.06	0.38 ± 0.02	0.12	0.80	0.34
	Phe-Tyr	0.73 ± 0.01	0.79 ± 0.02	0.71 ± 0.08	0.75 ± 0.04	0.74 ± 0.01	0.78 ± 0.07	0.78 ± 0.03	0.77 ± 0.03	0.74 ± 0.02	0.70	0.83	0.95
EAAI		77.12± 0.70	77.45± 3.87	75.68± 8.18	71.52± 8.18	82.56±1.08[*]	79.83± 1.47	72.36 ± 3.54	70.88 ± 2.34	69.72±2.66[#]	0.30	0.46	0.43

Table S4. Effects of short-term depuration under saline conditions on major volatile compounds in muscle of *Carassius auratus gibelio.*

Relative Content (%)	D0S0	D5				D10			
		S0	S3	S6	S9	S0	S3	S6	S9
hexanal	6.85±0.88	7.82±2.24	8.75±2.79	8.38±6.79	9.89±2.11	7.80±2.07	8.30±6.36	8.21±1.54	9.20±1.34
4-ethylbenzaldehyde	1.79±0.63	1.70±0.58	1.55±0.03	1.21±0.50	1.40±0.34	0.59	1.26	1.87±0.32	0.76
5-hexyl-3,3-dimethylcyclopentene	4.35±1.91	-	6.44±1.09	1.73±0.14	8.50±1.58	7.36±0.90	3.46±2.10	-	-
2-Propionylfuran	0.73	0.66±0.39	0.99	0.86	-	1.10±0.70	0.95±0.62	-	0.75±0.22
methyl myristate	2.68±1.25	2.09±0.34	1.62±0.52	1.31±0.02	1.40±0.84	2.14±1.71	2.36±0.25	3.03±1.50	1.62±0.68
ethyl palmitate	5.30±1.90	3.30±0.72	2.15±0.34	12.07±9.13	1.55±0.18	3.85±0.30	2.99±1.13	5.08±1.11	3.29±2.34
methyl laurate	1.90±0.73	2.13	2.22±0.19	2.95±0.27	2.34±0.28	1.96±0.45	1.86±0.14	1.76±0.35	2.17±0.65
Methyl Palmitate	9.22±1.07	7.71	4.38±1.88	5.19±1.62	2.77±1.78	10.16±3.32	17.75±2.73	11.71±2.96	8.13±5.82
1,2-diphenoxyhexane	2.32±1.32	2.71±0.40	2.96±0.64	2.59±0.26	2.03±0.75	1.03	2.69±0.49	4.58	2.56±0.54

Note: “-”indicates not detected, absence of standard error denotes ≤2 detected replicates.
